# Supplementary material for: Who marries whom and intentions for second child: Using family decision-making power as mediator
Source: PLoS One. 2025 Jun 26;20(6):e0326733. doi: 10.1371/journal.pone.0326733 (PMC12201641; doi:10.1371/journal.pone.0326733)
Supplement: S1 Table — (DOCX) [file pone.0326733.s001.docx]

S1 Path Coefficients of the GSEM Model (Full)

|  | Model 1 | Model 2 | Model 3 | Model 4 | Model 5 | Model 6 |
| --- | --- | --- | --- | --- | --- | --- |
|  | Husband-dominated | Fertility intention | Jointly decided | Fertility intention | Wife-dominated | Fertility intention |
|  | Coefficients  (SE) | Coefficients  (SE) | Coefficients  (SE) | Coefficients  (SE) | Coefficients  (SE) | Coefficients  (SE) |
| Educational assortative mating (ref. low-education homogamy) | | | | | | |
| Hypergamy | 0.031**  (0.014) | 0.023**  (0.011) | 0.023*  (0.012) | 0.024**  (0.011) | -0.054***  (0.013) | 0.022**  (0.011) |
| Mid-education homogamy | -0.092***  (0.017) | 0.002  (0.013) | 0.030**  (0.014) | -0.000  (0.013) | 0.062***  (0.015) | 0.002  (0.013) |
| High-education homogamy | -0.086**  (0.027) | 0.087***  (0.020) | 0.058**  (0.022) | 0.085***  (0.020) | 0.029  (0.025) | 0.086***  (0.020) |
| Hypogamy | -0.165***  (0.018) | -0.015  (0.013) | 0.055***  (0.014) | -0.018  (0.013) | 0.109***  (0.016) | -0.015  (0.013) |
| Power distribution in household decision-making | | | | | | |
| Husband-dominated |  | 0.021**  (0.008) |  |  |  |  |
| Joint decision-making |  |  |  | 0.006  (0.010) |  |  |
| Wife-dominated |  |  |  |  |  | -0.030**  (0.010) |
| Age | -0.002**  (0.001) | -0.001  (0.001) | 0.001  (0.001) | -0.001  (0.001) | 0.001*  (0.001) | -0.001  (0.001) |
| Gender (ref. female) | | | | | | |
| Male | -0.019  (0.014) | 0.009  (0.011) | -0.010  (0.012) | 0.009  (0.011) | 0.029**  (0.013) | 0.010  (0.011) |
| *Hukou* status (ref. rural) | | | | | | |
| Urban | -0.085***  (0.014) | -0.016  (0.011) | 0.017  (0.011) | -0.018*  (0.011) | 0.067***  (0.013) | -0.016  (0.011) |
| Ethnicity (ref. non-Han) | | | | | | |
| Han | -0.027  (0.019) | 0.002  (0.014) | 0.033**  (0.016) | 0.001  (0.014) | -0.006  (0.017) | 0.001  (0.014) |
| Geographical region (ref. Eastern) | | | | | | |
| Central | -0.047***  (0.013) | 0.033**  (0.010) | -0.001  (0.010) | 0.032**  (0.010) | 0.049***  (0.012) | 0.034***  (0.010) |
| Western | 0.064***  (0.014) | 0.024**  (0.010) | -0.028**  (0.011) | 0.026**  (0.010) | -0.036**  (0.012) | 0.024**  (0.010) |
| Log of household income | 0.004  (0.006) | 0.004  (0.004) | 0.009*  (0.005) | 0.004  (0.004) | -0.12**  (0.005) | 0.004  (0.004) |
| Actual number of children | 0.047***  (0.007) | 0.118***  (0.005) | -0.012**  (0.006) | 0.119***  (0.005) | -0.035***  (0.006) | 0.118***  (0.005) |
| Wives’ economic dependency | 0.013  (0.008) | 0.006  (0.006) | 0.001  (0.007) | 0.007  (0.006) | -0.014  (0.008) | 0.006  (0.006) |
| Wives’ relative amount of housework | -0.086***  (0.019) | 0.011  (0.015) | -0.007  (0.016) | 0.009  (0.014) | 0.093  (0.017) | 0.012  (0.015) |
| N | 8 321 | 8 202 | 8 321 | 8 202 | 8 321 | 8 202 |
| Log Likelihood | -9023.202 | | -7266.047 | | -8164.093 | |
| AIC | 18112.4 | | 14598.09 | | 16394.19 | |
| BIC | 18344.28 | | 14829.97 | | 16626.06 | |
