# Supplementary material for: Who marries whom and intentions for second child: Using family decision-making power as mediator
Source: PLoS One. 2025 Jun 26;20(6):e0326733. doi: 10.1371/journal.pone.0326733 (PMC12201641; doi:10.1371/journal.pone.0326733)
Supplement: S2 Table — (DOCX) [file pone.0326733.s002.docx]

S2 Descriptive Statistics of the Variables (By Gender)

|  | Male | | Female | |
| --- | --- | --- | --- | --- |
| Variables | Count | Mean (SD) / % | Count | Mean (SD) / % |
| **Fertility intentions for a second child** |  |  |  |  |
| No | 706 | 16.90% | 677 | 16.82% |
| Yes | 3 471 | 83.10% | 3 348 | 83.18% |
| **Patterns of educational mating** |  |  |  |  |
| Hypergamy | 1 244 | 29.78% | 1 200 | 29.81% |
| Low-education homogamy | 1 270 | 30.40% | 1 208 | 30.01% |
| Mid-education homogamy | 759 | 18.17% | 737 | 18.31% |
| High-education homogamy | 254 | 6.08% | 248 | 6.16% |
| Hypogamy | 650 | 15.56% | 632 | 15.70% |
| **Power in household decision-making** |  |  |  |  |
| Husband-dominated | 2 192 | 52.48% | 2 213 | 54.98% |
| Joint decision-making | 811 | 19.42% | 762 | 18.93% |
| Wife-dominated | 1 174 | 28.11% | 1 050 | 26.09% |
| **Items to assess decision-making power** |  |  |  |  |
| Household budget allocation | 1 415 | 38.36% | 2 290 | 64.36% |
| Savings, investments, and insurance | 1 307 | 35.43% | 2 370 | 66.61% |
| Real estate purchase | 1 028 | 27.87% | 2 632 | 73.97% |
| Children’s education | 1 941 | 52.62% | 1 749 | 49.16% |
| High-priced consumer goods | 1 479 | 40.16% | 2 205 | 62.06% |
| **Age** | 4 177 | 38.72 (7.52) | 4 025 | 40.45(7.81) |
| ***Hukou* status** |  |  |  |  |
| Rural | 3 187 | 76.30% | 2 969 | 73.76% |
| Urban | 990 | 23.70% | 1 056 | 26.24% |
| **Ethnicity** |  |  |  |  |
| Non-Han | 394 | 9.43% | 345 | 8.57% |
| Han | 3 783 | 90.57% | 3 680 | 91.43% |
| **Geographical region** |  |  |  |  |
| Eastern | 1 786 | 42.76% | 1 727 | 42.91% |
| Central | 1 232 | 29.49% | 1 198 | 29.76% |
| Western | 1 159 | 27.75% | 1 100 | 27.33% |
| **Log of household income** | 4 177 | -0.03 (0.92) | 4025 | -0.01(0.95) |
| **Actual number of children** | 4 177 | 1.61 (0.83) | 4025 | 1.59(0.83) |
| **Wives’ economic dependency** | 4 177 | -0.23 (0.63) | 4025 | -0.25(0.62) |
| **Wives’ relative amount of housework** | 4 177 | 0.91 (0.16) | 4025 | 0.41(0.36) |
