# Supplementary material for: Who marries whom and intentions for second child: Using family decision-making power as mediator
Source: PLoS One. 2025 Jun 26;20(6):e0326733. doi: 10.1371/journal.pone.0326733 (PMC12201641; doi:10.1371/journal.pone.0326733)
Supplement: S3 Table — (DOCX) [file pone.0326733.s003.docx]

S1 Table Path Coefficients of the GSEM Model (Male Samples)

|  | Model 1 | Model 2 | Model 3 | Model 4 | Model 5 | Model 6 |
| --- | --- | --- | --- | --- | --- | --- |
|  | Husband-dominated | Fertility intention | Jointly decided | Fertility intention | Wife-dominated | Fertility intention |
|  | Coefficients  (SE) | Coefficients  (SE) | Coefficients  (SE) | Coefficients  (SE) | Coefficients  (SE) | Coefficients  (SE) |
| Hypergamy | 0.033  (0.020) | 0.049**  (0.015) | 0.024  (0.017) | 0.050**  (0.015) | -0.057**  (0.018) | 0.048**  (0.015) |
| Low-education homogamy | *Reference* | *Reference* | *Reference* | *Reference* | *Reference* | *Reference* |
| Mid-education homogamy | -0.087***  (0.024) | 0.007  (0.018) | 0.028  (0.019) | 0.005  (0.018) | 0.058**  (0.022) | 0.008  (0.018) |
| High-education homogamy | -0.092**  (0.039) | 0.101**  (0.029) | 0.042  (0.031) | 0.100**  (0.029) | 0.050  (0.035) | 0.101**  (0.029) |
| Hypogamy | -0.162***  (0.025) | -0.011  (0.019) | 0.055**  (0.020) | -0.014  (0.019) | 0.107***  (0.022) | -0.010  (0.019) |
| Husband-dominated |  | 0.019  (0.012) |  |  |  |  |
| Joint decision-making |  |  |  | 0.012  (0.015) |  |  |
| Wife-dominated |  |  |  |  |  | -0.0033**  (0.013) |
| Control variables | *Controlled* | *Controlled* | *Controlled* | *Controlled* | *Controlled* | *Controlled* |
| N | 4 103 | 4 025 | 4 103 | 4 025 | 4 103 | 4 025 |
| Log Likelihood | -4420.588 | | -3554.245 | | -3973.392 | |
| AIC | 8903.177 | | 7170.491 | | 8008.783 | |
| BIC | 9099.08 | | 7366.394 | | 8204.687 | |
