# Supplementary material for: Who marries whom and intentions for second child: Using family decision-making power as mediator
Source: PLoS One. 2025 Jun 26;20(6):e0326733. doi: 10.1371/journal.pone.0326733 (PMC12201641; doi:10.1371/journal.pone.0326733)
Supplement: S4 Table — (DOCX) [file pone.0326733.s004.docx]

S2 Table Mediation Effects (Male Samples)

|  | Wife-dominated | Total effects |
| --- | --- | --- |
| Hypergamy | **0.0019**  **[0.0004, 0.0045]** | **0.0499**  **[0.0212, 0.0778]** |
| Low-education homogamy | *Reference* | *Reference* |
| Mid-education homogamy | **-0.0019**  **[-0.0051, -0.0003]** | 0.0056  [-0.0305, 0.0401] |
| High-education homogamy | -0.0017  [-0.0061, 0.0003] | **0.0997**  **[0.0368, 0.1564]** |
| Hypogamy | **-0.0036**  **[-0.0077, -0.0008]** | -0.0134  [-0.0526, 0.0239] |
