# Supplementary material for: Who marries whom and intentions for second child: Using family decision-making power as mediator
Source: PLoS One. 2025 Jun 26;20(6):e0326733. doi: 10.1371/journal.pone.0326733 (PMC12201641; doi:10.1371/journal.pone.0326733)
Supplement: S5 Table — (DOCX) [file pone.0326733.s005.docx]

S3 Table Path Coefficients of the GSEM Model (Female Samples)

|  | Model 1 | Model 2 | Model 3 | Model 4 | Model 5 | Model 6 |
| --- | --- | --- | --- | --- | --- | --- |
|  | Husband-dominated | Fertility intention | Jointly decided | Fertility intention | Wife-dominated | Fertility intention |
|  | Coefficients  (SE) | Coefficients  (SE) | Coefficients  (SE) | Coefficients  (SE) | Coefficients  (SE) | Coefficients  (SE) |
| Hypergamy | 0.030  (0.020) | -0.002  (0.015) | 0.021  (0.016) | -0.001  (0.015) | -0.050**  (0.018) | -0.002  (0.015) |
| Low-education homogamy | *Reference* | *Reference* | *Reference* | *Reference* | *Reference* | *Reference* |
| Mid-education homogamy | -0.097***  (0.024) | -0.004  (0.018) | 0.031  (0.019) | -0.006  (0.018) | 0.066**  (0.022) | -0.004  (0.018) |
| High-education homogamy | -0.081**  (0.038) | 0.074**  (0.029) | 0.074**  (0.031) | 0.723**  (0.029) | 0.008  (0.035) | 0.072**  (0.029) |
| Hypogamy | -0.168***  (0.025) | -0.017  (0.019) | 0.057**  (0.020) | -0.021  (0.019) | 0.111***  (0.023) | -0.018  (0.019) |
| Husband-dominated |  | 0.023**  (0.011) |  |  |  |  |
| Joint decision-making |  |  |  | 0.000 |  |  |
| Wife-dominated |  |  |  |  |  | -0.028**  (0.013) |
| Control variables | *Controlled* | *Controlled* | *Controlled* | *Controlled* | *Controlled* | *Controlled* |
| N | 4 218 | 4 177 | 4 218 | 4 177 | 4 218 | 4 177 |
| Log Likelihood | -4587.99 | | -3698.172 | | -4173.7818 | |
| AIC | 9237.981 | | 7458.344 | | 8409.564 | |
| BIC | 9434.742 | | 7655.104 | | 8606.324 | |
