# Supplementary material for: Who marries whom and intentions for second child: Using family decision-making power as mediator
Source: PLoS One. 2025 Jun 26;20(6):e0326733. doi: 10.1371/journal.pone.0326733 (PMC12201641; doi:10.1371/journal.pone.0326733)
Supplement: S6 Table — (DOCX) [file pone.0326733.s006.docx]

S4 Table Mediation Effects (Female Samples)

|  | Husband-dominated | Total effects | Wife-dominated | Total effects |
| --- | --- | --- | --- | --- |
| Hypergamy | 0.0006  [-0.0001, 0.0025] | -0.0009  [-0.0274, 0.0265] | **0.0014**  **[0.0002, 0.0038]** | -0.0009  [-0.0274, 0.0265] |
| Low-education homogamy | *Reference* | *Reference* | *Reference* | *Reference* |
| Mid-education homogamy | **-0.0022**  **[-0.0052, -0.0002]** | -0.0057  [-0.0410, 0.0274] | **-0.0019**  **[-0.0049, -0.0003]** | -0.0057  [-0.0409, 0.0275] |
| High-education homogamy | **-0.0019**  **[-0.0055, 0.0002]** | **0.0723**  **[0.0120, 0.1316]** | -0.0002  [-0.0028, 0.0019] | **0.0722**  **[0.0113, 0.1314]** |
| Hypogamy | **-0.0038**  **[-0.0082, -0.0000]** | -0.0213  [-0.0584, 0.0167] | **-0.0031**  **[-0.0069, -0.0004]** | -0.0213  [-0.0583, 0.0167] |
