# Supplementary material for: Who marries whom and intentions for second child: Using family decision-making power as mediator
Source: PLoS One. 2025 Jun 26;20(6):e0326733. doi: 10.1371/journal.pone.0326733 (PMC12201641; doi:10.1371/journal.pone.0326733)
Supplement: Attachment — Submitted filename: 5.11 response to the reviewers.docx [file pone.0326733.s010.docx]

**Reviewer #1:**

*Thanks for revising the manuscript based on my earlier comments. However, the discussion strands where you have meticulously cited previous authors in the concluding section have to be removed. Ensure that the concluding section pivots solely on inferences made from the key results that marries well with the stated study's objectives. Thanks!*

Response:

Thank you for your suggestions! All citations to previous authors have been removed from the conclusion.

**Reviewer #3:**

*The Authors have addressed all comments. The study is an impactful one and I recommend the manuscript for possible publication.*

Response:

Thank you very much.
