# Supplementary material for: Who marries whom and intentions for second child: Using family decision-making power as mediator
Source: PLoS One. 2025 Jun 26;20(6):e0326733. doi: 10.1371/journal.pone.0326733 (PMC12201641; doi:10.1371/journal.pone.0326733)
Supplement: Attachment — Submitted filename: Peer Reviewer Comments.pdf [file pone.0326733.s007.pdf]

Who marries whom and intentions for second child: using family decision-making power as mediator

Recommendation – ACCEPT With Minor Revision

Peer Reviewer Comments

Reviewer Assessment of the entire Manuscript

The title "Who Marries Whom and Intentions for a Second Child: Using Family Decision-Making Power as a Mediator" is highly relevant in the context of contemporary family dynamics and demographic trends. In recent years, there has been a growing interest in understanding how assortative mating—where individuals pair based on similarities in education, socio-economic status, or cultural background—affects various aspects of family life. This trend is particularly important because it influences household decision-making processes and ultimately impacts fertility intentions.

The concept of family decision-making power plays a crucial role in understanding fertility decisions. In many societies, traditional gender roles have been evolving, leading to more egalitarian household structures. However, disparities in education and income between spouses can still result in one partner having more influence over important family decisions. By examining who holds the decision-making power in the context of marital patterns, the study aims to shed light on how these dynamics affect the intention to have a second child.

Global trends in the 21st century, such as increased female workforce participation, urbanization, and economic uncertainties, have contributed to changing fertility patterns. Many countries are experiencing lower birth rates and shifts in family size preferences. Understanding the motivations and barriers that couples face when deciding to expand their families is essential for addressing these demographic challenges. The study's focus on the intention to have a second child is particularly relevant, as it provides insights into the factors that influence family planning decisions in the modern era.

The research methodology, which includes latent class analysis and generalized structural equation modeling, allows for a comprehensive examination of the relationships between educational attainment, decision-making power, and fertility intentions. By identifying the mediation effects of decision-making power, the study can reveal the mechanisms through which marital patterns influence fertility decisions. This approach provides a deeper understanding of the complex interplay between socio-economic factors and family dynamics.

The findings of this research have significant implications for public policy and social programs. By uncovering the factors that drive fertility intentions, policymakers can design interventions that support families in making informed decisions about family size. For instance, promoting gender equality and supporting women in achieving higher educational attainment can help balance decision-making power within

households. In addition, programs that provide economic support and childcare options can alleviate some of the barriers to having a second child, particularly in societies facing declining birth rates.

In summary, this study reflects a study that is deeply relevant to understanding contemporary issues in family dynamics and fertility trends. By examining these themes in the context of 21st-century global trends, the research can offer valuable insights for policymakers, sociologists, and demographers, ultimately contributing to the development of effective policies and programs that support family well-being.

My Comments:

1. Abstarct: Your abstract is quite comprehensive and informative. Here are a few suggestions to improve clarity and readability:

Abstract:

Interest in exploring fertility intentions, decisions, and the actual number of children through the perspective of assortative mating has been increasing; however, the mechanisms linking these variables remain unclear. Existing studies have shown that gaps in socio-economic resources between spouses shape intra-household decision-making patterns. Individuals who have the final-say power over homemaking exhibit more bargaining power in family fertility decisions. Based on the 2014 China Family Panel Studies, this research used latent class analysis to obtain the intra-household decision-making variable. A generalized structural equation model was built to examine this potential mediator.

The findings reveal that differences in couples' educational attainment are a key aspect in assessing "who" is in charge of the household. The desire for a second child was greater if husbands had the final say. Participants in marriages where wives held the decision-making power reported a lower willingness to have a second child. The mediation effects of "husband-dominated" or "wife-dominated" decision-making were confirmed in hypergamous marriages. Indirect-only mediating effects were found in mid-educated homogamous partnerships and hypogamous marriages. Suppression effects were present in educational homogamy among highly educated individuals.

2. Reduce the keywords to the following suggested ones:

Keywords: Educational assortative mating, fertility intention, household decision-making power, latent class analysis, generalized structural equation modeling

3. How do educational and socio-economic differences between spouses influence household decision-making power and fertility intentions?

- Exploring this question can help to clarify the mechanisms through which assortative mating impacts family dynamics and fertility decisions.

4. What role do cultural and societal norms play in shaping decision-making power within households and fertility intentions?

   - Understanding the influence of cultural contexts can provide a more comprehensive view of how these factors affect family planning.

5. How does the balance of decision-making power within households affect the intention to have a second child in different demographic groups (e.g., urban vs. rural, different age groups)?

   - Investigating these variations can offer insights into how different populations experience and respond to these dynamics.

6. What are the short-term and long-term impacts of decision-making power dynamics on fertility intentions and outcomes?

   - This question can help to identify patterns and trends over time, offering a deeper understanding of the causal relationships.

7. How do existing family policies and economic conditions influence decision-making power and fertility intentions?

   - Examining the external factors that affect household dynamics can provide valuable information for policymakers.

8. To what extent do gender roles and expectations influence the mediation effects of decision-making power on fertility intentions?

   - Investigating this can shed light on the gender-specific aspects of decision-making and how they impact family planning.

9. How do personal beliefs and values regarding family size and child-rearing influence the decision-making power and fertility intentions of spouses?

   - Understanding the personal dimensions can add depth to the analysis of household dynamics.

10. What are the implications of your findings for social and family policy development?

    - Considering the practical applications of your research can enhance its relevance and impact.

11. Separate the Discussion section from the conclusion section.

12. What are the strengths and limitations of this study?

13. After the Discussion section, can you include a sub-heading on "Implications of the findings"

14. Ask for a Professional English editor to address all grammatical errors and lexis structure of your manuscript.

15. Kindly check your references and see if they are fully citied in the main content of your research. Kindly check and revisit them all.

By addressing these questions, you can delve deeper into the complex relationships between marital patterns, decision-making power, and fertility intentions, ultimately enhancing the robustness and relevance of your research.
